# Functional Independence of Taiwanese Children with Osteogenesis Imperfecta

**DOI:** 10.3390/jpm12081205

**Published:** 2022-07-24

**Authors:** Yu-Min Syu, Chung-Lin Lee, Chih-Kuang Chuang, Huei-Ching Chiu, Ya-Hui Chang, Hsiang-Yu Lin, Shuan-Pei Lin

**Affiliations:** 1 Department of Pediatrics, Far Eastern Memorial Hospital, New Taipei City 22021, Taiwan; b101098040@tmu.edu.tw; 2 Division of Genetics and Metabolism, Department of Pediatrics, MacKay Memorial Hospital, Taipei 10449, Taiwan; clampcage@yahoo.com.tw (C.-L.L.); g880a01@mmh.org.tw (H.-C.C.); wish1001026@gmail.com (Y.-H.C.); 3 Department of Rare Disease Center, MacKay Memorial Hospital, Taipei 10449, Taiwan; 4 Institute of Clinical Medicine, National Yang-Ming Chiao-Tung University, Taipei 11221, Taiwan; 5 Division of Genetics and Metabolism, Department of Medical Research, MacKay Memorial Hospital, Taipei 10449, Taiwan; mmhcck@gmail.com; 6 College of Medicine, Fu-Jen Catholic University, Taipei 24205, Taiwan; 7 Department of Childhood Care and Education, MacKay Junior College of Medicine, Nursing and Management, Taipei 11260, Taiwan; 8 Department of Medicine, MacKay Medical College, New Taipei City 25245, Taiwan; 9 Department of Medical Research, China Medical University Hospital, China Medical University, Taichung 40402, Taiwan; 10 Department of Pediatrics, MacKay Memorial Hospital, No. 92, Sec. 2, Chung-Shan North Road, Taipei 10449, Taiwan; 11 Department of Infant and Child Care, National Taipei University of Nursing and Health Sciences, Taipei 11219, Taiwan

**Keywords:** independent living, osteogenesis imperfecta, Taiwan, WeeFIM

## Abstract

Osteogenesis imperfecta (OI) is a group of rare genetic disorders that affect bone formation. Patients with OI present mainly with increased bone fragility and bone deformities. Twenty-seven Taiwanese children between 2 and 21 years of age with OI and their parents were recruited at MacKay Memorial Hospital from January 2013 to December 2019. We used the Functional Independence Measure for Children (WeeFIM) questionnaire to assess the functional independence of the children and describe any functional limitations or additional burden of daily care. Out of a potential score of 126, the mean total WeeFIM score was 113.7. There was a statistically significant difference between the scores of type I, type III and type IV OI (121.88 [SD 7.01] vs. 80.8 [SD 26.25] vs. 119.17 [SD 10.89]; *p* < 0.001). There were no statistically significant differences between the scores in different age groups, the male and female participants, and patients with pathogenic variants in *COL1A1* and *COL1A2*. The mean scores for the self-care, mobility, and cognition domains were 48.78 (maximum 56, mean quotient 91.14%), 30.44 (maximum 35, mean quotient 87.12%), and 34.44 (maximum 35, mean quotient 99.05%), respectively. The best performance was in the cognition domain (mean quotient 99.05%), and the worst was in the mobility domain (mean quotient 87.12%). There were no statistically significant correlations between WeeFIM scores and age, or age when symptoms began. The total WeeFIM score and 13 subscores for the self-care and mobility domains were all positively correlated with body height (*p* < 0.01). The correlation was lowest for bowel and walking/wheelchair tasks, and the highest for bathing and dressing-upper tasks. For tasks in bathing, over 40% of the patients needed help. For tasks in the cognition domain, most patients required no help. For the Taiwanese children with OI, some support and supervision were required for self-care and mobility tasks, and the functional independence in these two domains was correlated with body height and disease types. The WeeFIM questionnaire may be a useful tool to assess the functional strengths and weaknesses of children with OI.

## 1. Introduction

Osteogenesis imperfecta (OI) is a group of rare genetic disorders caused mostly by pathogenic variants in the *COL1A1* and *COL1A2* genes, inherited in an autosomal dominant manner. These two genes encode the alpha chains of collagen type 1, which is a main part of connective tissues that support the whole body, including the bones. The pathogenic variants in these genes cause defects in bone formation, and patients with OI present mainly with increased bone fragility, bone deformities, and other connective-tissue manifestations such as discoloration of the sclera, and soft teeth.

The revised Nosology and Classification of Genetic Skeletal Disorders identifies five clinical forms of OI [1]: non-deforming with persistently blue sclera (OI type I), perinatal lethal form (OI type II), progressively deforming type (OI type III), moderate form (OI type IV), and with calcification of the interosseous membranes and/or hypertrophic callus (OI type V). OI type I has the mildest phenotype, while patients with OI type III are most severely affected, suffering from frequent fractures, short stature, and restricted mobility.

A major concern for parents is the function and quality of life of their children. Because of the systemic nature of OI, the children may develop symptoms in multiple systems and defects in mobility and function. While specific impairments such as compromised joint range of motion and muscle strength in children with OI persist, functional abilities may improve over time [2,3]. Treatment should focus on optimal functioning of the child and family [4]. A multidisciplinary team, as well as social welfare and healthcare services, are required to provide integrated healthcare programs [5,6,7]. In Taiwan, “The Rare Disease and Orphan Drug Act” was implemented on 9 February 2000, and the rare disease case reporting database was also established in 2000 accordingly. When a diagnosis of OI is confirmed, the patients are registered in the database after being referred by a professional. Upon registration, the health authority offers counseling on a national level regarding medical, psychological, activity, social and educational aspects. Medications such as bisphosphonates are covered by the National Health Insurance program and given to Taiwanese OI patients when needed. As bisphosphonate therapy attenuates the frequency of fractures and improves the functional outcomes and quality of life in OI patients [7,8,9,10,11,12], the early administration of bisphosphonates may give these children unique features. It is therefore important for pediatricians to identify the functional strengths and weaknesses in specific tasks of these children and provide individualized care that can improve their quality of life. This information will not only provide insights into the functional prognosis for their parents, but also be of value for policy makers and service planners in terms of rehabilitation, vocation, long-term care, and accommodation.

The Functional Independence Measure for Children (WeeFIM) questionnaire is a convenient tool which is used to obtain information directly relevant to functional outcomes [13], and it has been modified for use in Chinese children [14]. In this study, we used the WeeFIM questionnaire to assess functional independence in Taiwanese children with OI between 2 and 21 years of age. Given the importance of understanding the impact of OI on functional independence, we aimed to quantify the range of functional performance of Taiwanese children with OI using the WeeFIM questionnaire, elucidate the associated factors, and describe any functional limitations or additional burden of daily care.

## 2. Methods

### 2.1. Study Population

Twenty-seven children between 2 and 21 years of age with OI and their parents were recruited at MacKay Memorial Hospital from January 2013 to December 2019. The parents and children completed the WeeFIM questionnaire at the clinic. The ethics committee of the hospital approved the study protocol, and all of the participants or their parents provided written informed consent.

The patients’ basic profiles and genotypes were recorded along with clinical features, including disease subtypes, body height, surgical history, age when symptoms began, and age at starting bisphosphonates. The age when symptoms began was defined as the age at which the patients had a first fracture or observed bony structural change that raised the suspicion of OI, such as bowed legs. The patients with a prenatal diagnosis of OI were excluded in the analysis on age when symptoms began and other parameters. The age at starting bisphosphonates was defined as the age at which the patient received the first bisphosphonates treatment, either by an oral or intravenous route. The timing of initiating bisphosphonates was determined by the clinicians, and mostly depended on the clinical severity and the willingness of the patients and their family.

This study was approved by the Institutional Review Board of MacKay Memorial Hospital. Patients aged 18 years and older signed a consent form, whereas those younger than 18 years signed an assent form. The parents also signed a consent form for their own participation, and if their child was under 18 years of age, a parental consent form.

### 2.2. WeeFIM Questionnaire

A Chinese version of the WeeFIM questionnaire [14,15,16] was used to estimate the functional independence of the enrolled children. The WeeFIM questionnaire was designed for primary caregivers to record their child’s abilities directly, and in 2002, Wong et al. translated the questionnaire into Chinese. It has been used to estimate functional independence in children from 6 months to 7 years of age, and up to 21 years of age in those with developmental disabilities [17,18,19,20].

The questionnaire includes 18 items categorized into three domains: self-care, mobility, and cognition. The self-care domain comprises eight items (eating, grooming, bathing, dressing-upper, dressing-lower, toileting, bladder, and bowel); the mobility domain comprises five items (chair transfer, toilet transfer, tub transfer, walking, and stairs); and the cognition domain also comprises five items (comprehension, expression, social interaction, problem-solving, and memory).

The response to each item is scored from 1 to 7 (1, total assistance required; 2, maximal contact assistance or prompting with 25–49% performance effort; 3, moderate contact assistance or prompting with 50–74% performance effort; 4, minimal contact assistance or prompting with >75% performance effort; 5, supervision, setup, or standby prompting; 6, modified independence using an assistive device, or not safe or timely performance; and 7, complete independence without a helper or device, safe, and timely performance) [21] (Table 1).

Scores from 1–5 indicate that the child is dependent and needs help in performing daily activities, while scores from 6–7 indicate independence with no help required. The self-care, mobility, and cognition domain scores range from 8–56, 5–35, and 5–35, respectively, and the total score ranges from 18–126 [22].

We calculated the total functional and domain functional quotients using age-based normal ratings (expressed as %) for Chinese children aged less than 7 years [14]. For those aged 7 years or older, the total rating norms was 126, including 56, 35, and 35 for self-care, mobility, and cognition, respectively.

### 2.3. Statistical Analysis

Descriptive statistics were performed and the results were presented as median (interquartile range, IQR) unless otherwise indicated. The normality test was performed using the Jarque-Bera test for scores. Because of the broad age range, the 27 children were stratified into 0–5, 6–10, 11–15, and 16–21 years old age groups for the evaluation of functional performance. The patients’ WeeFIM scores were compared to the normative Chinese children [14] with quotients (%) calculated. The differences of score quotients among the four age groups and three disease types were analyzed using one-way ANOVA. Pearson’s correlation coefficient (r) was used to determine the relationships among age, age when symptoms began, body height, and the 18 WeeFIM subscores. The WeeFIM scores were analyzed with body height as a continuous variable using linear regression analysis, sex (male or female), and disease-causing genes (*COL1A1* or *COL1A2* mutations) as class variables using the Student’s *t*-test. The WeeFIM scores of the same patients at two different time points were analyzed using the paired *t*-test. IBM SPSS Statistics software version 28.0 (IBM Corp., Armonk, NY, USA) was used to perform the statistical analysis. Statistical significance was set at *p* < 0.05.

## 3. Results

According to the “Statistical Report of Rare Disease Confirmed Cases in Taiwan, December 2019” published by the Health Promotion Administration, Ministry of Health and Welfare, Taiwan, there were a total of 369 confirmed OI patients nationwide by the end of the study period. Among these patients, 114 were under follow-up or receiving treatment at MacKay Memorial Hospital, which accounted for over 30% of all Taiwanese patients with OI. We recruited 27 (64%) of the 42 children with OI between 2 years and 21 years of age being followed at MacKay Memorial Hospital at the time of the study. Six of these 27 children (22%) had repeated WeeFIM questionnaire data after three years of follow-up. Fifteen children not willing to participate or with poor adherence were not enrolled.

Of the 27 children enrolled in this study, 12 were males and 15 were females. Their median age at enrollment was 11 years and 10 months, and seven patients were enrolled above the age of 18 years (three with type I, three with type III and one with type IV OI). The participants’ OI was confirmed by molecular studies, except for one patient. Eighteen children had *COL1A1* mutations (67%), eight had *COL1A2* mutations (30%) and one had neither *COL1A1* nor *COL1A2* mutations but a clinical diagnosis of OI type III. The age when symptoms began was recorded in 20 children, and two children were diagnosed prenatally by a positive family history of OI. Twenty-three children (85%) received bisphosphonate treatment, with a mean age at initiation of 58.1 months. Most of those children had mild flu-like symptoms in their first and second courses of bisphosphonate therapy, while the following courses were smooth and without adverse effect relevant to functional independence. Four children with OI type I did not receive bisphosphonate therapy based on their mild phenotype, with functional quotients of 100%, 100%, 96.8% and 97.6%, respectively.

The number of fractures were recorded in 25 patients and the number of surgical interventions in 21 patients. The details of the number of fractures, surgical interventions and casting durations were recorded in Table S1. The surgical details were recorded for 18 children; out of these patients, 12 had OI type I, 2 had type III and 4 had type IV. The median number of fractures were two and nine in children with OI type I and IV respectively while the data was not available for children with OI type III. The median number of surgical interventions were zero and one in children with OI type I and IV respectively. Most patients with OI type I had suffered a fracture less than four times, while those with OI type III had suffered from a fracture more than ten times.

The normality test rejected the null hypothesis of normal distributed data. The descriptive statistics were handled using median and interquartile range, and significance of differences between groups were calculated using quotients (%) of each of the subscores compared to normative Chinese children.

The total WeeFIM scores of the enrolled children ranged from 53 to 126 (median 124) with a mean quotient of 92.6% compared to normative data for Chinese children. The total, mean, and median scores, mean quotient, and interquartile range for each domain for the four age groups are shown in Table 2, and for the three disease types in Table 3. All age groups include at least one patient with type III OI, except for age group 2.

**Figure 1:**
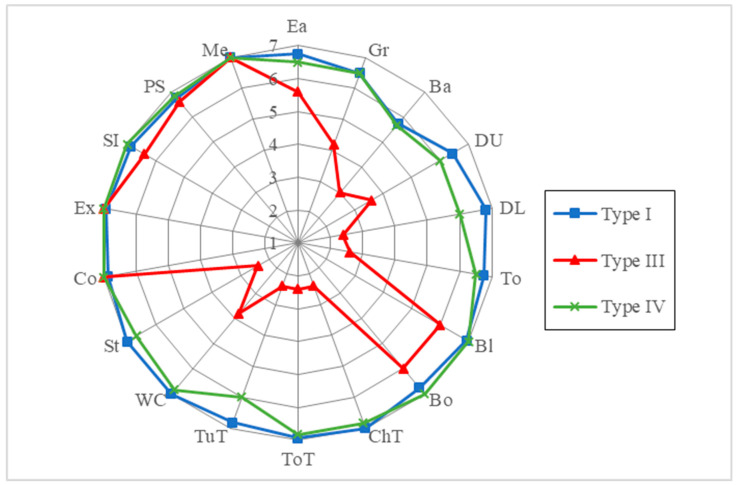
WeeFIM profiles of the study participants stratified by clinical type of OI.

**Figure 2:**
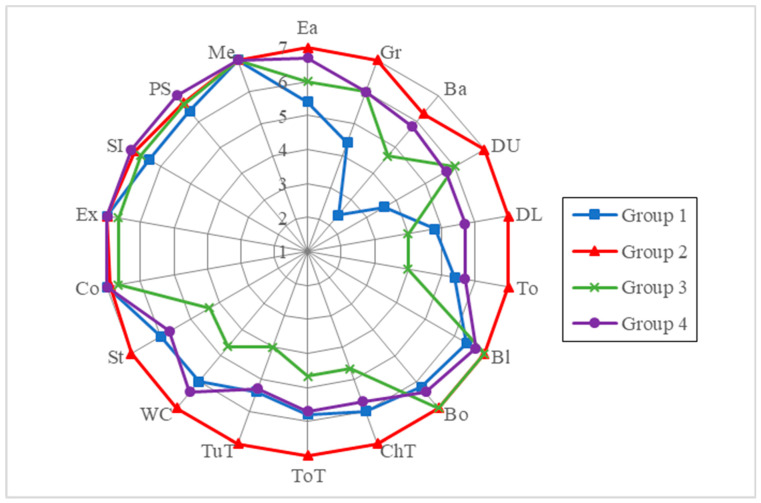
WeeFIM profiles of the study participants stratified by age group.

The mean scores for the self-care, mobility, and cognition domains were 48.8 (maximum 56, mean quotient 91.1%), 30.4 (maximum 35, mean quotient 87.1%), and 34.4 (maximum 35, mean quotient 99.1%), respectively. The median scores for the self-care, mobility, and cognition domains were 56, 35, and 35, respectively. The mean quotient of total WeeFIM scores of the patients with different disease types and the corresponding self-care and mobility scores were significantly different, while the corresponding cognition scores were not. Children with OI type III had the lowest scores in self-care, mobility and total domains compared to the other types of OI. Overall, the best performance was in the cognition domain (mean quotient 99.1%), and the worst was in the mobility domain (mean quotient 87.1%). According to the WeeFIM profiles of the study participants stratified by age and type of OI (Figure 1 and Figure 2), the overall weakest performance was in bathing.

**Figure 3:**
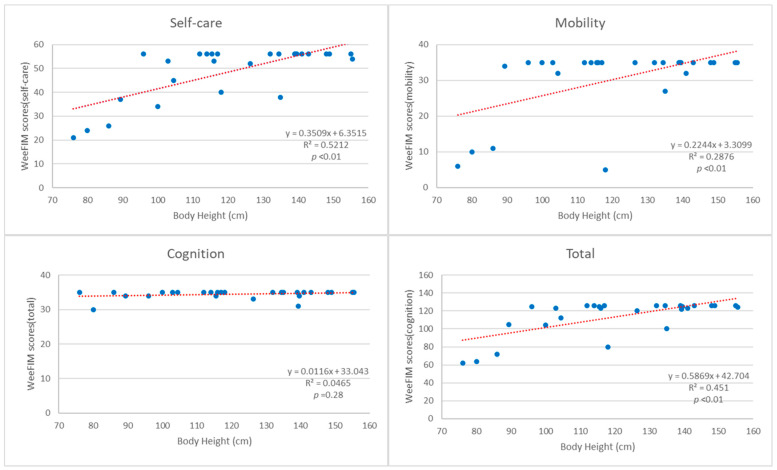
Regression analysis between WeeFIM scores and body height. Blue dots: subscores and body height of each patient; red dotted lines: regression curves for each subscores and body heights.

The total WeeFIM score and 13 subscores for the self-care and mobility domains were all positively correlated with body height (*p* < 0.01) (Table S2 and Figure 3).

The correlation was lowest for bowel and walking/wheelchair tasks, and highest for bathing and dressing-upper tasks (Table S3). There were no statistically significant correlations between WeeFIM scores and age, or age when symptoms began (Table S2). WeeFIM scores and subscores were not significantly different, while body height increased significantly between the two time points, in the six children with repeated evaluations (Table 4).

The total WeeFIM scores of the patients with *COL1A1* (median 123 [IQR11.75]) and *COL1A2* (median 125 [IQR 21.25]) gene mutations and the corresponding self-care (56 and 55), mobility (35 and 35), and cognition (35 and 35) scores were not significantly different. There were no significant differences in total WeeFIM scores between the boys (median 125 [IQR4.5]) and girls (median 123 [IQR26]) and the corresponding self-care (56 and 53), mobility (35 and 35), and cognition (35 and 35) scores.

The WeeFIM scores for each of the three domains of the children requiring help, requiring supervision or requiring no help are summarized in Table 5. For tasks in bathing, over 40% of the patients needed help. However, for tasks in the cognition domain, most patients required no help.

The mean subscores of each task according to patients in each disease type group and age group are shown as a radar chart in Figure 1 and Figure 2. Patients with type I and type IV OI had mild functional impairment, mostly in bathing (5.8 and 5.7, respectively). Patients with type III OI had generally affected functions in both self-care and mobility domains, with the worst subscores of 2.4 out of 7 in bed/chair/wheelchair transfer, toilet transfer, tub/shower transfer and stairs tasks. Figure 2 demonstrates a persistence of specific impairments in bathing tasks in age groups 1–4 (2.4, 6.3, 4.7, and 5.8 respectively).

## 4. Discussion

In this study, we used the WeeFIM questionnaire to analyze disabilities in cognition, self-care, and mobility, and to provide data on the range of functional performance across these three domains, in a group of children with OI between 2 and 21 years of age. Overall, 96.3% of the children did not require help with cognitive functioning, compared with 77.8% who did not require help with mobility, and 59.3% who did not require help with self-care. These results are consistent with previous studies [23,24,25]. WeeFIM total scores have been shown to differ significantly between disease type groups, as clinical severity is highly correlated with functional outcomes in mobility and self-care domains [23,26]. In this study, the patients with OI type III had the most severe phenotypes, and only one-third of the mean scores in the mobility domain compared to normative Chinese children. The lowest WeeFIM subscores in the children with OI type III were in dressing-lower limbs, chair transfer, toilet transfer and tub transfer tasks (Figure 1), which are highly correlated to mobility of the lower limbs. In addition, the item with the highest need for help (40.7%) among the study population was bathing, which involves functioning of both upper and lower extremities.

Previous studies have reported that upper-extremity deformities affect the performance of basic self-care tasks and impact mobility, transfers, and ambulation, especially in children with severe OI [27]. Moreover, patients with OI have a low muscle size [12,28], lower limb muscle weakness [12,29,30,31,32,33,34], restricted joint range of motion [32,35], pain [36,37] and fear of fractures when bearing weight [36,38]; all of which could cause obstacles in accomplishing these daily tasks and have a great impact on the functional independence of these patients.

Unlike the normative group, in the children with OI, the total WeeFIM scores and subscores in the three domains were not significantly correlated with age, and there were no significant differences among the different age groups. In contrast, the need for help for specific tasks was highly consistent among the different age groups, which is consistent with previous studies [2]. While 40.7% of the study group required help in bathing, two of the nine (22.2%) patients in age group 4 (16 to 21 years) still needed help with bathing. In addition, in the six children who had repeated WeeFIM questionnaire data, the total scores and subscores in all three domains did not significantly improve after 3 years, while the body height increased significantly. This shows the persistency of impaired functions throughout childhood and adolescence in children with OI.

The WeeFIM total score and 13 subscores for the self-care and mobility domains were positively correlated with body height (*p* < 0.05). The correlation between WeeFIM scores and body height was independent of disease severity, as the significance was consistent within the disease type groups (Figure S1). The height measurements in the children with OI in this study are consistent with the growth patterns observed in other studies [39,40,41,42]. The etiology for growth deficiency in OI is multifactorial. Factors that may affect body height, such as spinal deformities, recurrent long bone fractures, and bone deformities [43], can also contribute to functional impairments. However, height can also be affected in individuals without significant bone deformities [39,44]. In addition, the unresponsiveness of osteoblasts to normal growth factors [40] and excessive transforming growth factor-beta (TGF-β) signaling [45] may contribute to short stature, without impairing the functions of patients in daily activities. Our results address the importance of body height in children with OI as a correlating factor for functional outcomes in self-care and mobility domains.

Several measurement tools have been used in evaluation of functional outcomes in studies on children with OI [3,6,11,23]. Among those tools, PEDI (Pediatric Evaluation of Disability Inventory) and WeeFIM are most frequently used and both had been validated in Chinese children with disabilities [14,46]. The PEDI instrument, composed of 197 questions, is more time-consuming and costly in the setting of an outpatient department in Taiwan, while the WeeFIM instrument is more convenient and showed reliability in our previous studies regarding functional independence in Taiwanese children with rare diseases [18,19].The WeeFIM is a reliable instrument to evaluate the functional status of children with disabilities. It was first validated in American children [47], and then used by Wong et al. [14] and others [13] in similar studies. In a study conducted in Hong Kong, the Chinese language version of the WeeFIM that was developed by Wong et al. [14] obtained results consistent with earlier reports in Japanese children [15]. The WeeFIM has been proven in different cultures and languages to be a good tool to assess the strengths and weaknesses of participants with disabilities, however it cannot replace other psychological tests that evaluate adaptation, intelligence, and communication.

We found that the children with OI in the present study had relatively poor mobility function and self-care skills with preserved cognitive function. The patients with OI type IV usually achieved independence in self-care activities and general mobility, whereas those with OI type III had lower scores in both the mobility and self-care domains. These results may help in designing early interventions to foster self-care and independent living.

This study has several limitations. First, because of the rarity of OI, our sample size was small, while presumably 20% of the pediatric OI population in Taiwan were enrolled. We interpret the results carefully due to the small sample number, while the proportion should be representative enough. Due to the heterogeneity of our patients, we have calculated the quotient of each patient compared to the normative Chinese children, and conducted the statistical reanalysis using quotient instead of mean scores, and used median and IQR in the descriptive analysis. Second, the ability of the children to walk was scored by the parents and verified by studying health care records in the WeeFIM questionnaire. Furthermore, as the initial version of this questionnaire was not in Chinese, the English translation may include important nuances that were not recognized. The translation should be validated in each population to accurately define the norms. The records of the 27 children included complete medical histories on their macrocephaly, hydrocephalus, developmental delay and hearing problems. Out of the 27 patients, 18 of them had surgical details with different fracture numbers, surgical methods and varied casting duration. Because of the complexity of surgical courses and complications, most of the surgical details were not available in patients with OI type III. The heterogeneity of surgical courses would affect the outcome in the OI patients. Due to the limitation of the study design, some detailed surgical records were not available, and the sample size was too small to delineate the effect of surgical courses. To investigate the effects of these medical conditions on self-care and motor functioning in children with OI, larger and more comprehensive studies are needed. Besides, we repeated the WeeFIM questionnaire in only six children with a follow-up period of three years. Some of the patients did not receive repeated evaluation because they were aged over 21 years after the follow up periods; most of the patient with OI type I denied re-evaluation because their functional status did not change over time and their parents considered it not necessary to answer again. We interpreted the results from the small number of patients with caution and used the data only as supportive data to other results. A more comprehensive longitudinal analysis is necessary to assess functional outcomes over time.

## 5. Conclusions

For the Taiwanese children with OI in this study, some support and supervision were required for self-care and mobility tasks, and the functional independence in these two domains was correlated with body height and disease type. The WeeFIM questionnaire may be a useful tool to assess the functional strengths and weaknesses of children with OI.

## Figures and Tables

**Table 1:** Scores and items of the WeeFIM questionnaire.

**Levels**
Independent
7. Complete independence (timely, safe)
6. Modified independence (assistive device/not safe or timely)
Modified dependence
5. Supervision
4. Minimal assistance (subject > 75%)
3. Moderate assistance (subject = 50–74%)
Complete dependence
2. Maximal assistance (subject = 25–49%)
1. Total assistance (subject = 0–24%)
**Items**
Self-care
1. Eating (Ea)
2. Grooming (Gr)
3. Bathing (Ba)
4. Dressing-upper (DU)
5. Dressing-lower (DL)
6. Toileting (To)
7. Bladder (Bl)
8. Bowel (Bo)
Mobility
9. Bed/Chair/Wheelchair transfer (ChT)
10. Toilet transfer (ToT)
11. Tub/Shower transfer (TuT)
12. Walk/Wheelchair (WC)
13. Stairs (St)
Cognition
14. Comprehension (Co)
15. Expression (Ex)
16. Social interaction (SI)
17. Problem-solving (PS)
18. Memory (Me)

**Table 2:** WeeFIM scores of the children with osteogenesis imperfecta by age group.

	Group 1	Group 2	Group 3	Group 4	Total
Age (years)	2.0–5.9	6.0–10.9	11–15.9	16–21	2–21
*n*	5	10	3	9	27
Self-care score					*p* = 0.28
Range	24–53	52–56	38–56	13–56	13–56
Median score	37	56	40	56	56
Mean quotient (%) ^a^	89.64	99.29	79.76	86.7	91.14
Interquartile range	11	0	9	2	13.5
Mobility score					*p* = 0.206
Range	10–35	35	5–35	5–35	5–35
Median score	34	35	27	35	35
Mean quotient (%) ^a^	84.17	100	63.8	82.22	87.12
Interquartile range	3	0	15	3	2
Cognition score					*p* = 0.09
Range	30–35	33–35	31–35	35	30–35
Median score	35	35	35	35	35
Mean quotient (%) ^a^	100	98.57	96.19	100	99.05
Interquartile range	1	1	2	0	0.5
Total score					*p* = 0.211
Range	64–123	120–126	80–122	53–126	53–126
Median score	105	125.5	100	126	124
Mean quotient (%) ^a^	93.06	99.29	79.89	89.15	92.6
Interquartile range	8	1	21	3	17.5

^a^ Comparisons to normative data for Chinese children.

**Table 3:** WeeFIM scores of the children with osteogenesis imperfecta by disease type.

	Type I	Type III	Type IV	Total
Mean age (years)	11.2	15.3	11.1	11.9
*n*	16	5	6	27
Self-care score				*p* < 0.001
Range	34–56	13–56	38–56	13–56
Mean score	52.69	33.4	51.17	48.78
Median score	56	26	56	56
Mean quotient (%) ^a^	98.98	63.07	93.6	91.14
Standard deviation	6.87	14.59	7.8	11.3
Interquartile range	3	16	8.25	13.5
Mobility score				*p* < 0.001
Range	32–35	5–35	27–35	5–35
Mean score	34.75	13.4	33.17	30.44
Median score	35	10	35	35
Mean quotient (%) ^a^	99.46	38.45	94.76	87.12
Standard deviation	0.78	12.34	3.25	9.74
Interquartile range	0	5	2.25	2
Cognition score				*p* = 0.486
Range	31–35	30–35	34–35	30–35
Mean score	34.44	34	34.83	34.44
Median score	35	35	35	35
Mean quotient (%) ^a^	98.57	100	99.52	99.05
Standard deviation	1.09	2.24	0.41	1.25
Interquartile range	1	0	0	0.5
Total score				*p* < 0.001
Range	104–126	53–126	100–126	53–126
Mean score	121.88	80.8	119.17	113.7
Median score	124.5	68	125.5	124
Mean quotient (%) ^a^	99.16	67.03	96.43	92.6
Standard deviation	7.01	26.25	10.89	20.33
Interquartile range	3.25	16	10.75	17.5

^a^ Comparisons to normative data for Chinese children. Type I, non-deforming OI with persistently blue sclera; type III, progressively deforming OI; type IV, moderate OI; OI, osteogenesis imperfecta.

**Table 4:** Comparison of WeeFIM scores and body height between two time points.

Number of Patients			6	
Mean Age of First Evaluation (Months)			113.8	
Age Range (Months)			53–252	
	Mean-Pre	Mean-Post	Coefficient	*p* Value
Self-care score	41.2	50	0.84	0.053
Mobility score	28	30	1	0.1
Cognition score	32.5	35	0.42	0.41
Total score	102	115	0.9	0.052
Body height	103	113	0.9	0.044 *

* statistically significant at *p* = 0.05 level.

**Table 5:** Scores of individual WeeFIM tasks grouped into help, supervision, and no help categories.

	Requiring Help (1–4 Points)		Requiring Supervision (5 Points)		Requiring no Help (6–7 Points)	
Task	*n*	%	*n*	%	*n*	%
Eating	4	14.8	0	0.0	23	85.2
Grooming	6	22.2	0	0.0	21	77.8
Bathing	11	40.7	0	0.0	16	59.3
Dressing-upper	7	25.9	0	0.0	20	74.1
Dressing-lower	7	25.9	0	0.0	20	74.1
Toileting	6	22.2	0	0.0	21	77.8
Bladder	1	3.7	1	3.7	25	92.6
Bowel	1	3.7	2	7.4	24	88.9
Chair transfer	4	14.8	0	0.0	23	85.2
Toilet transfer	4	14.8	0	0.0	23	85.2
Tub transfer	7	25.9	0	0.0	20	74.1
Walking	3	11.1	0	0.0	24	88.9
Stairs	4	14.8	1	3.7	22	81.5
Comprehension	0	0.0	0	0.0	27	100.0
Expression	0	0.0	0	0.0	27	100.0
Social interaction	1	3.7	0	0.0	26	96.3
Problem-solving	0	0.0	1	3.7	26	96.3
Memory	0	0.0	0	0.0	27	100.0

## Data Availability

Not applicable.

## Supplementary Materials

The following supporting information can be downloaded at: https://www.mdpi.com/article/10.3390/jpm12081205/s1, Figure S1: Regression analysis between WeeFIM scores and body height in the patients with OI type I; Table S1: Detailed surgical profiles of patients; Table S2: Pearson correlation coefficients between WeeFIM scores and variables; Table S3: Pearson correlation coefficients between body height and WeeFIM scores and subscores.

## Abbreviations

OI	osteogenesis imperfecta
WeeFIM	Functional Independence Measure for Children
IQR	interquartile range

## References

[1] Mortier G.R., Cohn D.H., Cormier-Daire V., Hall C., Krakow D., Mundlos S., Nishimura G., Robertson S., Sangiorgi L., Savarirayan R. (2019). Nosology and classification of genetic skeletal disorders: 2019 revision. Am. J. Med. Genet. A.

[2] Engelbert R.H., Beemer F.A., van der Graaf Y., Helders P.J. (1999). Osteogenesis imperfecta in childhood: Impairment and disability—A follow-up study. Arch. Phys. Med. Rehabil..

[3] Engelbert R.H., Uiterwaal C.S., Gerver W.J., van der Net J.J., Pruijs H.E., Helders P.J. (2004). Osteogenesis imperfecta in childhood: Impairment and disability. A prospective study with 4-year follow-up. Arch. Phys. Med. Rehabil..

[4] Nijhuis W., Verhoef M., van Bergen C., Weinans H., Sakkers R. (2022). Fractures in Osteogenesis Imperfecta: Pathogenesis, Treatment, Rehabilitation and Prevention. Children.

[5] Marom R., Rabenhorst B.M., Morello R. (2020). Osteogenesis imperfecta: An update on clinical features and therapies. Eur. J. Endocrinol..

[6] Montpetit K., Palomo T., Glorieux F.H., Fassier F., Rauch F. (2015). Multidisciplinary Treatment of Severe Osteogenesis Imperfecta: Functional Outcomes at Skeletal Maturity. Arch. Phys. Med. Rehabil..

[7] Arshad F., Bishop N. (2021). Osteogenesis imperfecta in children. Bone.

[8] Sousa T., Bompadre V., White K.K. (2014). Musculoskeletal functional outcomes in children with osteogenesis imperfecta: Associations with disease severity and pamidronate therapy. J. Pediatr. Orthop..

[9] Garganta M.D., Jaser S.S., Lazow M.A., Schoenecker J.G., Cobry E., Hays S.R., Simmons J.H. (2018). Cyclic bisphosphonate therapy reduces pain and improves physical functioning in children with osteogenesis imperfecta. BMC Musculoskelet. Disord..

[10] Dwan K., Phillipi C.A., Steiner R.D., Basel D. (2016). Bisphosphonate therapy for osteogenesis imperfecta. Cochrane Database Syst. Rev..

[11] Seikaly M.G., Kopanati S., Salhab N., Waber P., Patterson D., Browne R., A Herring J. (2005). Impact of alendronate on quality of life in children with osteogenesis imperfecta. J. Pediatr. Orthop..

[12] Veilleux L.N., Trejo P., Rauch F. (2017). Muscle abnormalities in osteogenesis imperfecta. J. Musculoskelet. Neuronal Interact..

[13] Ottenbacher K.J., Msall M.E., Lyon N. (2000). Measuring developmental and functional status in children with disabilities. Pediatr. Phys. Ther..

[14] Wong V., Wong S., Chan K., Wong W. (2002). Functional Independence Measure (WeeFIM) for Chinese children: Hong Kong Cohort. Pediatrics.

[15] Wong S.S., Wong V.C. (2007). Functional Independence Measure for Children: A comparison of Chinese and Japanese children. Neurorehabilit. Neural Repair..

[16] Du Q., Salem Y., Liu H.H., Zhou X., Chen S., Chen N., Yang X., Lialng J., Sun K. (2017). A home-based exercise program for children with congenital heart disease following interventional cardiac catheterization: Study protocol for a randomized controlled trial. Trials.

[17] Wong V., Chung B., Hui S., Fong A., Lau C., Law B., Lo K., Shum T., Wong R. (2004). Cerebral palsy: Correlation of risk factors and functional performance using the Functional Independence Measure for Children (WeeFIM). J. Child Neurol..

[18] Lee C.L., Lin H.Y., Chuang C.K., Chiu H.C., Tu R.Y., Huang Y.H., Hwu W.-L., Tsai F.-J., Chiu P.-C., Niu D.-M. (2019). Functional independence of Taiwanese patients with mucopolysaccharidoses. Mol. Genet. Genom. Med..

[19] Lee C.L., Lin H.Y., Tsai L.P., Chiu H.C., Tu R.Y., Huang Y.H., Chien Y.-H., Lee N.-C., Niu D.-M., Chao M.-C. (2018). Functional independence of Taiwanese children with Prader-Willi syndrome. Am. J. Med. Genet. A.

[20] Lin H.Y., Chuang C.K., Chen Y.J., Tu R.Y., Chen M.R., Niu D.M., Lin S.-P. (2016). Functional independence of Taiwanese children with Down syndrome. Dev. Med. Child Neurol..

[21] Sperle P.A., Ottenbacher K.J., Braun S.L., Lane S.J., Nochajski S. (1997). Equivalence reliability of the functional independence measure for children (WeeFIM) administration methods. Am. J. Occup. Ther..

[22] Lin H.Y., Lin S.P., Lin H.Y., Hsu C.H., Chang J.H., Kao H.A., Hung H.-Y., Peng C.-C., Lee H.-C., Chen M.-R. (2012). Functional independence of Taiwanese children with VACTERL association. Am. J. Med. Genet. A.

[23] Engelbert R.H.H., Custers J.W.H., van der Net J., van der Graaf Y., Beemer F.A., Helders P.J.M. (1997). Functional Outcome in Osteogenesis Imperfecta: Disability Profiles Using the PEDI. Pediatr. Phys. Therapy.

[24] Vanz A.P., van de Sande Lee J., Pinheiro B., Zambrano M., Brizola E., da Rocha N.S., Schwartz I.V.D., Pires M.M.D.S., Félix T.M. (2018). Health-related quality of life of children and adolescents with osteogenesis imperfecta: A cross-sectional study using PedsQL. BMC Pediatr..

[25] Constantino C.S., Krzak J.J., Fial A.V., Kruger K.M., Rammer J.R., Radmanovic K., Smith P.A., Harris G.F. (2019). Effect of Bisphosphonates on Function and Mobility Among Children With Osteogenesis Imperfecta: A Systematic Review. JBMR Plus..

[26] Engelbert R.H., Uiterwaal C.S., Gulmans V.A., Pruijs H., Helders P.J. (2000). Osteogenesis imperfecta in childhood: Prognosis for walking. J. Pediatr..

[27] Amako M., Fassier F., Hamdy R.C., Aarabi M., Montpetit K., Glorieux F.H. (2004). Functional analysis of upper limb deformities in osteogenesis imperfecta. J. Pediatr. Orthop..

[28] Palomo T., Glorieux F.H., Schoenau E., Rauch F. (2016). Body Composition in Children and Adolescents with Osteogenesis Imperfecta. J. Pediatr..

[29] Veilleux L.N., Pouliot-Laforte A., Lemay M., Cheung M.S., Glorieux F.H., Rauch F. (2015). The functional muscle-bone unit in patients with osteogenesis imperfecta type I. Bone.

[30] Gremminger V.L., Phillips C.L. (2021). Impact of Intrinsic Muscle Weakness on Muscle-Bone Crosstalk in Osteogenesis Imperfecta. Int. J. Mol. Sci..

[31] Pavone V., Mattina T., Pavone P., Falsaperla R., Testa G. (2017). Early Motor Delay: An Outstanding, Initial Sign of Osteogenesis Imperfecta Type 1. J. Orthop. Case Rep..

[32] Brizola E., Staub A.L., Felix T.M. (2014). Muscle strength, joint range of motion, and gait in children and adolescents with osteogenesis imperfecta. Pediatr. Phys. Ther..

[33] Veilleux L.N., Lemay M., Pouliot-Laforte A., Cheung M.S., Glorieux F.H., Rauch F. (2014). Muscle anatomy and dynamic muscle function in osteogenesis imperfecta type I. J. Clin. Endocrinol. Metab..

[34] Veilleux L.N., Darsaklis V.B., Montpetit K., Glorieux F.H., Rauch F. (2017). Muscle Function in Osteogenesis Imperfecta Type IV. Calcif. Tissue Int..

[35] Badhyal S., Dhole S.R., Gopinathan N.R., Dhillon M.S., Dhiman V., Jayal A.D., Prasad J. (2019). Kinetic and Kinematic Analysis of Gait in Type IV Osteogenesis Imperfecta Patients: A Comparative Study. Indian J. Orthop..

[36] Caudill A., Flanagan A., Hassani S., Graf A., Bajorunaite R., Harris G., Smith P. (2010). Ankle strength and functional limitations in children and adolescents with type I osteogenesis imperfecta. Pediatr. Phys. Ther..

[37] Zack P., Franck L., Devile C., Clark C. (2005). Fracture and non-fracture pain in children with osteogenesis imperfecta. Acta Paediatr..

[38] Dahan-Oliel N., Oliel S., Tsimicalis A., Montpetit K., Rauch F., Dogba M.J. (2016). Quality of life in osteogenesis imperfecta: A mixed-methods systematic review. Am. J. Med. Genet. A.

[39] Germain-Lee E.L., Brennen F.S., Stern D., Kantipuly A., Melvin P., Terkowitz M.S., Shapiro J.R. (2016). Cross-sectional and longitudinal growth patterns in osteogenesis imperfecta: Implications for clinical care. Pediatr. Res..

[40] Aglan M.S., Zaki M.E., Hosny L., El-Houssini R., Oteify G., Temtamy S.A. (2012). Anthropometric measurements in Egyptian patients with osteogenesis imperfecta. Am. J. Med. Genet. A.

[41] Barber L.A., Abbott C., Nakhate V., Do A.N.D., Blissett A.R., Marini J.C. (2019). Longitudinal growth curves for children with classical osteogenesis imperfecta (types III and IV) caused by structural pathogenic variants in type I collagen. Genet. Med..

[42] Graff K., Syczewska M. (2017). Developmental charts for children with osteogenesis imperfecta, type I (body height, body weight and BMI). Eur. J. Pediatr..

[43] Jain M., Tam A., Shapiro J.R., Steiner R.D., Smith P.A., Bober M.B., Hart T., Cuthbertson D., Krischer J., Mullins M. (2019). Growth characteristics in individuals with osteogenesis imperfecta in North America: Results from a multicenter study. Genet. Med..

[44] Lund A.M., Muller J., Skovby F. (1999). Anthropometry of patients with osteogenesis imperfecta. Arch. Dis. Child..

[45] Grafe I., Yang T., Alexander S., Homan E.P., Lietman C., Jiang M.M., Bertin T., Munivez E., Chen Y., Dawson B. (2014). Excessive transforming growth factor-beta signaling is a common mechanism in osteogenesis imperfecta. Nat. Med..

[46] Chen K.L., Hsieh C.L., Sheu C.F., Hu F.C., Tseng M.H. (2009). Reliability and validity of a Chinese version of the Pediatric Evaluation of Disability Inventory in children with cerebral palsy. J. Rehabil. Med..

[47] Msall M.E., DiGaudio K., Duffy L.C., LaForest S., Braun S., Granger C.V. (1994). WeeFIM. Normative sample of an instrument for tracking functional independence in children. Clin. Pediatr. (Phila).

