# Supplementary material for: Functional Independence of Taiwanese Children with Osteogenesis Imperfecta
Source: J Pers Med. 2022 Jul 24;12(8):1205. doi: 10.3390/jpm12081205 (PMC9394323; doi:10.3390/jpm12081205)
Supplement: Supplementary file 1 [file jpm-12-01205-s001.zip › jpm-1779613-SI.pdf]

Supplementary Tables

Table S1. Detailed surgical profiles of patients

Patient No.	OI type	No. of fractures	No. of surgery	Total casting time	Surgical methods
1	1	1	0	1m1d	Closed reduction
2	1	4	2	N/A	CRIF
3	1	4	1	5m5d	CRIF
4	1	1	0	N/A	-
5	1	9	N/A	N/A	N/A
6	1	2	1	2m	N/A
7	1	2	0	N/A	-
8	1	3	N/A	Long-term bracing	N/A
9	1	3	1	N/A	N/A
10	1	0	0	-	-
11	1	0	0	-	-
12	1	2	0	4m6d	-
13	1	3	1	N/A	CRIF
14	1	2	0	2m23d	-
15	1	0	0	-	-
16	1	2	1	1m11d	CRIF
17	3	N/A	N/A	N/A	N/A
18	3	20+	N/A	N/A	N/A
19	3	15	11	N/A	CRIF, ORIF(Fassier-Duval nail), corrective osteotomy
21	3	N/A	N/A	N/A	N/A
22	3	13	8	15m19d	ORIF(Fassier-Duval nail), corrective osteotomy
23	4	9	N/A	N/A	N/A
24	4	2	1	1m	Closed reduction
25	4	9	1	4m1d	CRIF
26	4	10+	10+	N/A	CRIF, ORIF
27	4	1	1	N/A	ORIF
28	4	10+	10+	N/A	N/A

CRIF, Closed reduction-internal fixation; ORIF, open reduction internal fixation.

Table S2. Pearson correlation coefficients between WeeFIM scores and variables

**Variables**	**Age**		**Age when symptoms began**		**Body height**	
	r	*p value*	*r*	*p*	*r*	*p*
Self-care score	0.45	0.82	0.35	0.13	0.72**	<0.01
Quotient (%)	-0.25	0.21	0.32	0.17	0.56**	<0.01
Mobility score	-0.19	0.36	0.28	0.23	0.54**	<0.01
Quotient (%)	-0.19	0.34	0.28	0.23	0.53	<0.01
Cognition score	0.22	0.22	0.00	0.998	0.22	0.28
Quotient (%)	-0.12	0.55	-0.22	0.36	-0.14	0.49
Total score	-0.05	0.81	0.32	0.17	0.67**	<0.01
Quotient (%)	-0.26	0.19	0.27	0.24	0.53**	<0.01

**. statistically significant at p=0.01 level.

Table S3. Pearson correlation coefficients between body height and WeeFIM scores and subscores

**Task**	***r***
Self-care score	0.72**
Quotient (%)	0.557**
Eating	.487**
Grooming	.627**
Bathing	.712**
Dressing-upper	.695**
Dressing-lower	.612**
Toileting	.584**
Bladder	.588**
Bowel	.421*
Mobility score	0.536**
Quotient (%)	0.529**
Chair transfer	.545**
Toilet transfer	.573**
Tub transfer	.494**
Walking	.417*
Stairs	.532**
Cognition score	0.216
Quotient (%)	-0.137
Total score	0.672**
Quotient (%)	0.529**

**. statistically significant at p=0.01 level.
*. statistically significant at p=0.05 level.

Supplementary Figures

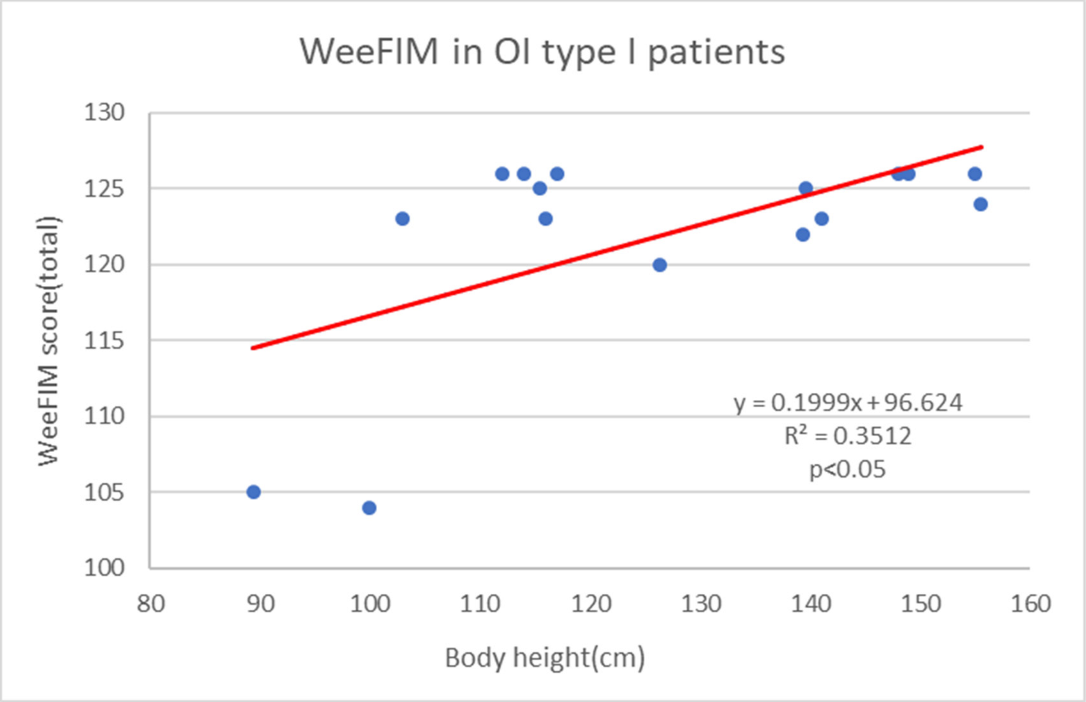

Figure S1. Regression analysis between WeeFIM scores and body height in the patients with OI type I
